# Hypertensive crisis associated with high dose soy isoflavone supplementation in a post-menopausal woman: a case report [ISRCTN98074661]

**DOI:** 10.1186/1472-6874-5-9

**Published:** 2005-06-23

**Authors:** Andrea M Hutchins, Imogene E McIver, Carol S Johnston

**Affiliations:** 1 Department of Nutrition, Arizona State University, 7001 East Williams Field Road, Mesa, AZ 85212, USA

## Abstract

**Background:**

Isoflavones are gaining popularity as alternatives to hormone replacement therapy. However, few guidelines exist to inform the public as to an appropriate dose. This case involves a postmenopausal woman who experienced a hypertensive crisis while consuming a high-dose isoflavone supplement as part of a research protocol.

**Case Presentation:**

The participant was part of a placebo-controlled crossover trial to investigate the potential synergism of the antioxidant activity of soy isoflavones and vitamin C. Upon entry into the study, this healthy, well-nourished, normotensive postmenopausal woman (51 years old), consumed the first of four randomly assigned treatments (500 mg vitamin C plus 5 mg/kg body weight soy isoflavones). During this treatment, the participant's systolic blood pressure spiked to a recorded 226/117 mmHg, necessitating medical intervention and discontinuation of study participation. Two plausible mechanisms for this hypertensive crisis are discussed.

**Conclusion:**

Due to the availability and increasing popularity of soy supplements, practitioners should be aware of the potential side effects associated with their use. Practitioners counseling clients who are consuming soy isoflavone supplements should advise them that elevated blood pressure may be a potential side-effect to consider and monitor.

## Background

In recent years, isoflavones have increased in popularity as an alternative to conventional hormone replacement therapy for the relief of hot flashes and other symptoms associated with menopause. Currently, isoflavones are available as tablets, capsules, powders (particularly soy protein powders), drinks and bars [1] as well as a component of traditional soy foods. Typically, supplements provide 25–100 mg total isoflavones if consumed according to package directions [1]. Yet, due to the increasing variety of soy foods in the marketplace, consumers can easily consume 100 mg or more of total isoflavones each day from the diet alone. Although soy foods have been available for millennia, isoflavone supplements are relatively new and few drug/supplement or nutrient/supplement interactions have been identified [1]. However, consumers should be advised to use caution when taking isoflavone supplements because the potential for unidentified interactions does exist. This case study presents a postmenopausal woman who experienced an isoflavone/nutrient interaction, which resulted in a hypertensive crisis requiring medical intervention.

## Case presentation

A 51-year-old postmenopausal non-Hispanic white woman was treated for a hypertensive crisis at a regional medical center in eastern Arizona. She had complained of symptoms for one week prior to admission, including light-headedness, headaches, and high blood pressure by self-measurement. Ten days prior to admission, the patient had been enrolled in a university-sponsored research trial designed to investigate the extent to which vitamin C and soy isoflavones, as supplements to a habitual diet, could provide antioxidant effects by reducing *in vivo *oxidative damage to cells, either alone or synergistically. During trial screening the patient reported typically consuming soy or soy products twice a week; no regular alcohol consumption; no history of hypertension or cardiovascular disease (although there was a family history of mild hypertension); no current medical supervision or care for any chronic health problems; no current use of over-the-counter or prescription medications and a routine exercise pattern of three times a week for 30–60 minutes. The participant weighed 175 pounds (79.5 kg), stood 5'8" (1.73 m), with a body mass index of 26.7 kg/m^2^.

Early in the research trial, the patient was randomized to receive 500 mg vitamin C plus 5 mg/kg body weight soy isoflavones. On trial day 3, the patient reported to the investigators that she felt "odd" and "light-headed." At the time, this was not attributed to the study-related supplements because the participant reported experiencing infrequent headaches for the past 20 years. On trial days 6 and 7 of the treatment period, the participant had her blood pressure checked by an automated machine; the readings were in the range of 140–150/92–98 mmHg vs. her usual BP of 120/82 mmHg. Due to this unexpected occurrence, the investigators requested that she stop consuming the supplements and drop out of the study. The incident was reported the university's Institutional Review Board Research Compliance Office, and the research trial was allowed to continue. Unbeknownst to the investigators, the participant chose to ignore the request to discontinue the supplements and continued to take the supplements on trial days 8 and 9. On trial day 9 she found her BP to be 159/110 mmHg. That night, she experienced an intense headache, a feeling of anxiety, and difficulty sleeping. Around midday on trial day 10, she stopped by a regional medical center to have her BP checked by a medical professional before going hiking. At that time, her BP was 226/117 mmHg; she reported that "my head feels like it is going to explode" and she was admitted to the emergency room. Laboratory analyses, including a complete blood count, metabolic panel and thyroid stimulating hormone test, were all within normal limits. A CT scan of the head showed no abnormalities or intracranial hemorrhages and a 12 lead EKG showed a normal sinus rhythm. At this time, the participant reported to the physician a 20-year history of chronic headaches that had resolved with better sleep habits and a higher fluid intake. The participant was then given 20 mg of the alpha_1 _and beta-blocker labetalol HCl via intravenous infusion (see Table 1). Subsequent to administration of the medication, the participant's blood pressure slowly dropped below critical levels, but did not reach normal limits. She was dismissed from the emergency room after 3 1/2 hours with a prescription for the non-selective beta-blocker propranolol HCl (Inderal LA), 80 mg once a day. She was told to discontinue the supplements that she was taking for the research trial.

**Table 1:** Vital signs and pertinent events during emergency room admission

		Blood Pressure				
					
Date	Time	Systolic	Diastolic	Heart Rate	Respirations	Notes
March 10	1301	226	117	87	20	
	1310					12 lead EKG performed Blood drawn for laboratory analyses
	1334	203	115	75	18	
	1339	190	110	70	20	Taken to CT
	1345	178	112	78	16	
	1349	176	100	78	18	
	1354	177	111	71	12	
	1357	177	118	72	10	
	1359	187	106	70	18	Medicated via IV: Labetolol HCl 20 mg Pt. stated ↓ headache
	1404	162	96	67	11	
	1409	159	94	74	16	
	1414	169	111	72	15	
	1419	177	108	72	17	
	1424	181	104	73	27	
	1429	163	93	69	13	
	1434	169	97	69	15	
	1439	170	111	72	22	
	1444	162	97	84	14	
	1449	168	104	70	13	Pt. stated headache gone
	1454	171	104	65	12	
	1459	166	92	69	14	
	1504	190	106	74	18	
	1509	181	107	69	34	
	1514	177	106	76	13	
	1519	163	104	71	20	
	1524	178	108	70	14	
	1529	176	106	64	20	
	1534	168	102	89	9	
	1539	162	100	70	16	
	1544	168	102	68	14	
	1549	165	102	70	12	Medicated PO: Inderal LA 80 mg
	1618	179	117	69	N/A	
	1630					Discharged to home

The patient notified the trial investigator of the hypertensive event several days later. The hypertensive crisis was reported to the university's Institutional Review Board Research Compliance Office, and the research trial was allowed to continue with the stipulation that all participants submit to blood pressure monitoring weekly. Later that week, the participant's BP was measured by the primary investigator's staff and was still above normal limits. When the participant saw a cardiologist and her regular physician for further follow-up, no abnormalities in cardiac function, renal function or hormone levels were identified that could have led to the hypertensive crisis. The participant continued on antihypertensive medications for the next 12 months and was gradually able to decrease the dose of the medications over time.

## Discussion

One plausible explanation for the hypertensive crisis experienced by this participant is the inhibition of monoamine oxidase by the isoflavones (e.g., daidzin, daidzein) or their metabolites (e.g., equol). Rooke et al.[2] and Gao et al.[3] both reported that daidzin, the plant precursor of the mammalian metabolite daidzein, and some of its structural analogs can inhibit mitochondrial monoamine oxidase *in vitro*. Additionally, Dewar et al.[4] reported that equol, a mammalian metabolite of daidzein, was an effective inhibitor of rat liver monoamine oxidase *in vitro*. Since the soy isoflavone supplements used in the research trial consisted of 63% (178 mg aglycone units/g) genistein, 28% (79.1 mg aglycone units/g) daidzein and 9% (24.6 aglycone units/g) glycitein (percentages based on aglycone units), the daidzein in the supplement may have interacted with monoamine oxidase.

Monoamine oxidase is responsible for the deamination of monoamines, including serotonin, epinephrine, norepinephrine, dopamine and tyramine. Its inhibition will cause an increase in the blood levels of these compounds. Since tyramine acts as a vasoconstrictor, an increased tyramine level will cause an increase in blood pressure [5,6]. Review of the two-day food records recorded prior to the participant's entering the study in addition to dietary information obtained after the hypertensive event indicated the participant's normal diet typically contained multiple tyramine-containing foods. The participant confirmed that she had consumed several tyramine-containing foods during the study, including the day before and the day of her emergency room admission (Table 2). Thus, the high dose of supplemental isoflavones [397.5 mg isoflavones (aglycone units) containing approximately 111 mg daidzein (aglycone units)], in conjunction with her typical moderate to high tyramine diet, may have contributed to a monoamine oxidase inhibitor-type reaction. Although the studies by Rooke et al.[2], Gao et al. [3] and Dewar et al.[4] suggest such a reaction might be possible, we believe this is the first report published of a possible monoamine oxidase inhibitor reaction and subsequent blood pressure spike occurring *in vivo *due to intake of a soy isoflavone supplement.

**Table 2:** Participant's Dietary Intake

Dinner on Day 9, 5:30 p.m.
*Yoplait fat free yogurt–12 ounces Peanuts, salted–1/4 cup Navel orange–1 medium *Banana, ripe–1 medium *Avocado, ripe–1 small Potato chips–1 handful Jelly beans–1/4 cup *3 Musketeers bar–1/3 of bar Vanilla ice cream–1/2 cup

Breakfast on Day 10, 8:00 a.m.

*Coffee–21 ounces *Bacon–3 slices Eggs, scrambled–2 whole Toast–1 slice with ~1 teaspoon margarine

* signifies tyramine-containing foods

Foods from participant's Typical Diet containing tyramine or other pressor agents[5,6]

Balsamic vinegar–1–2 teaspoons daily Cheddar cheese–2–4 ounces daily Mozzarella cheese–1 ounce daily Yogurt–16 ounces daily Dried beans or legumes–1/2 cup daily Coffee–17–21 ounces daily Bananas–1 every other day Avocado–3 times/week Tamari sauce–1 tablespoon 2 times/week Swiss cheese–2 ounces/week Cured meats–1 time/week Raisins–2–3 times/month Spinach–2–3 times/month Blue Cheese–2–3 times/month Chocolate–occasionally

A second plausible explanation for the hypertensive crisis experienced by this participant is an imbalance in the renin-angiotensin system, an important regulator of blood pressure, due to the administration of the isoflavones. Isoflavones are known to bind to both the α and β estrogen receptors and exert weak estrogenic effects in vivo [7,8]. Because angiotensinogen production by the liver is modulated by estrogens, the assumed increase in the serum isoflavone concentrations due to the high isoflavone intake may have stimulated an estrogenic response, thereby increasing hepatic angiotensinogen production and release into the plasma [9-11]. Once cleaved by renin, angiotensinogen becomes angiotensin I which is rapidly converted to angiotensin II by the angiotensin-converting enzyme [12]. Angiotensin II acts on the outer layer of the zona glomerulosa of the adrenal cortex, converting corticosterone to aldosterone, which subsequently increases renal sodium reabsorption as well as extracellular fluid and blood volume resulting in an increase in blood pressure [12]. Thus, the high dose of supplemental isoflavones consumed by this participant may have caused an imbalance in the renin-angiotensin system, the end result of which was the hypertensive crisis that the participant experienced.

## Conclusion

Due to the availability and increasing popularity of soy supplements, practitioners should be aware of the potential side effects associated with their use. This case study reports two plausible reactions, a monoamine oxidase inhibitor-type reaction or an imbalance in the renin-angiotensin system, which may have occurred with consumption of a high-dose isoflavone supplement resulting in the participant experiencing a hypertensive crisis. Although this reaction occurred within the context of a research study, it is possible that similar reactions might occur in general population if the dosage guidelines listed on the soy isoflavone supplements are exceeded. Practitioners counseling clients who are consuming soy isoflavone supplements should advise them that elevated blood pressure may be a potential side-effect to consider and monitor.

## Competing interests

The author(s) declare that they have no competing interests.

## Authors' contributions

AH participated in the design and coordination of the study and drafted the manuscript. IM participated in the design of the study, participated in conducting the laboratory analyses, and helped to draft the manuscript. CJ participated in the design of the study, performed the statistical analyses, and helped to draft the manuscript.

## Pre-publication history

The pre-publication history for this paper can be accessed here:


